# Tumoral Calcinosis of the Knee With Secondary Infection in a Patient With Juvenile Dermatomyositis: Management Approach and Pathophysiological and Therapeutic Review

**DOI:** 10.7759/cureus.90008

**Published:** 2025-08-13

**Authors:** Brandon Eric Dkhar, Narendra Kumar, M C Shashidhar, Siddhartha S Basumatary, Vipul Pathak

**Affiliations:** 1 Central Institute of Orthopaedics, Vardhman Mahavir Medical College and Safdarjung Hospital, New Delhi, IND

**Keywords:** dystrophic calcification, juvenile dermatomyositis, multidisciplinary management, superadded infection, tumoral calcinosis

## Abstract

Tumoral calcinosis is a debilitating complication of juvenile dermatomyositis (JDM) that results from dystrophic calcification in inflamed soft tissues. Although often asymptomatic, it can occasionally be complicated by secondary infections, requiring both medical and surgical interventions. Here, we report a rare case of tumoral calcinosis with superadded infection in a child with JDM, along with a brief pathophysiological and therapeutic overview. An 11-year-old girl with a known history of JDM presented with fever and localized pain in the right knee. Examination revealed local warmth, mild joint restriction, and no other systemic signs. Imaging demonstrated periarticular soft tissue calcifications without osseous involvement. MRI and CT scans confirmed lobulated, calcified soft tissue masses with surrounding edema. The patient underwent surgical incision, drainage, and excision of accessible calcified nodules. Histopathology confirmed tumoral calcinosis with associated inflammation. Postoperative rehabilitation began two weeks after surgery, with favorable functional recovery. Calcinosis in JDM develops through chronic inflammation, metabolic imbalance, and impaired calcium clearance due to dysfunctional macrophages. Contributing factors include genetic susceptibility, immune complex deposition, and mitochondrial injury. Imaging, particularly MRI and CT, is critical for diagnosis and surgical planning. Management requires a multidisciplinary approach, including phosphate restriction, chelation therapy, immunosuppressive agents, and selective surgical excision. Emerging options such as antioxidants, including N-acetylcysteine, have shown promise in slowing disease progression. This case underscores the importance of early recognition and targeted therapy for tumoral calcinosis in JDM. Prompt, coordinated intervention can help prevent irreversible joint damage and improve the quality of life in affected children.

## Introduction

Tumoral calcinosis is an extremely rare inflammatory myopathy of childhood, with an unknown exact global incidence and fewer than 300 cases reported worldwide. Its prevalence is estimated at less than 1 in 1 million people. The condition is characterized by the deposition of calcium phosphate crystals in periarticular soft tissues, forming lobulated, calcified masses [1]. Despite its name, tumoral calcinosis is not a true neoplasm, as it lacks mitotic activity and proliferative potential. It can occur as a complication of various systemic and metabolic disorders, including connective tissue diseases such as juvenile dermatomyositis (JDM) [2].

In JDM, dystrophic calcification develops in 20%-40% of cases, often due to chronic inflammation and delayed initiation of therapy [3]. While tumoral calcinosis is typically asymptomatic and slow-growing, it may become clinically significant when complicated by secondary infection, leading to localized pain, swelling, fever, and functional impairment.

Diagnosis requires a combination of imaging modalities, biochemical testing, and histopathological confirmation. Management can be challenging and may involve both medical and surgical approaches.

This case report describes an 11-year-old girl with JDM who developed tumoral calcinosis complicated by secondary infection. It highlights the importance of maintaining a high index of suspicion, ensuring timely diagnosis, and implementing multidisciplinary care with individualized surgical planning.

## Case presentation

An 11-year-old girl with a known diagnosis of JDM, on continuous immunosuppressive therapy for the past two years, presented with acute-onset pain and swelling of the right knee, accompanied by low-grade fever persisting for two months. There was no history of trauma or similar symptoms in other joints.

On examination, localized warmth was noted over the right knee, with mild restriction in the range of motion. The patient remained ambulatory and was able to bear weight. No additional cutaneous manifestations were observed beyond those attributable to her underlying dermatomyositis.

**Figure 1:**
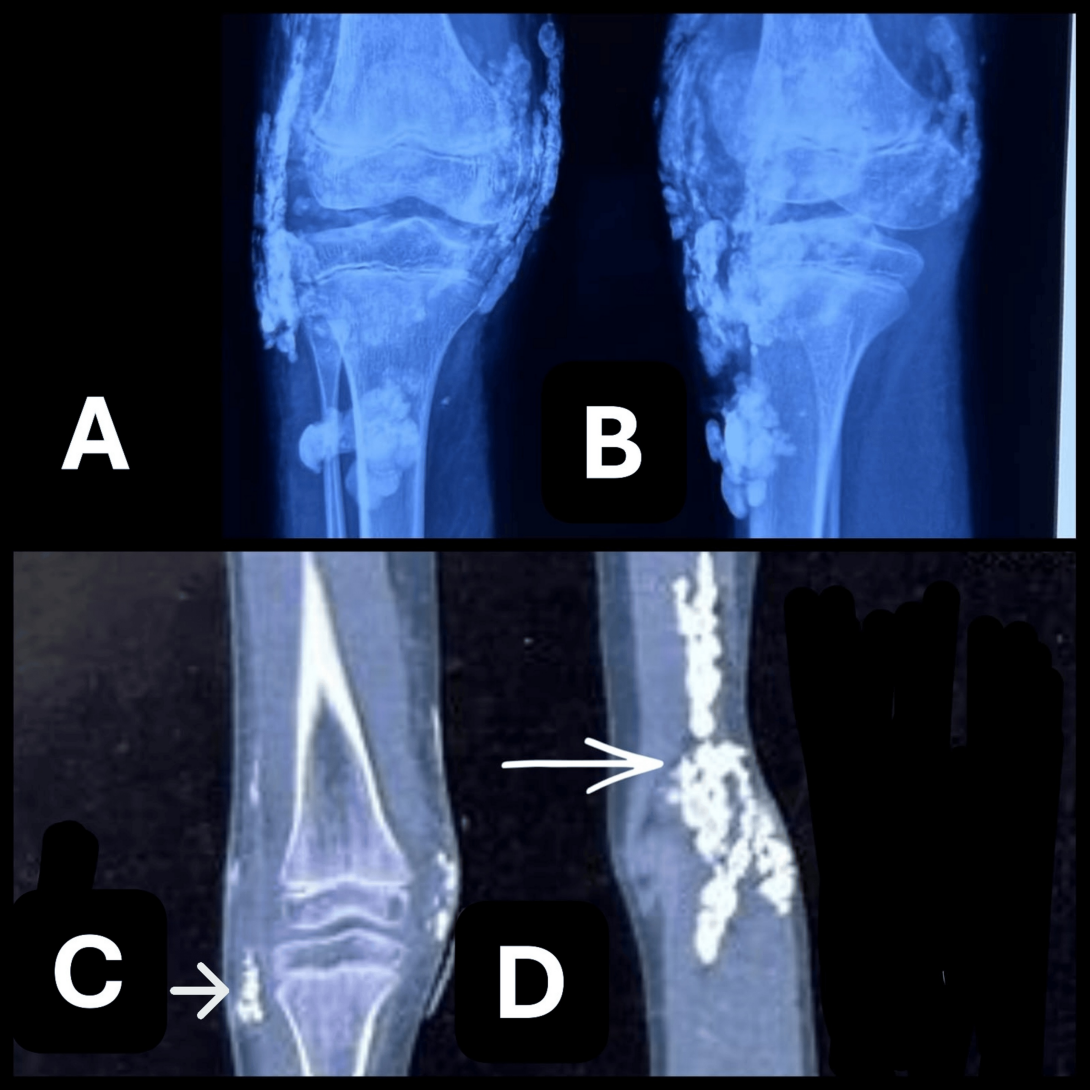
Dystrophic calcification (A) AP and (B) lateral radiographs of the left knee showing overall extensive calcification. Coronal cut computed tomography of the (C) right and (D) left knee showing calcification (white arrows).

Biochemical investigations showed normal serum calcium, phosphate, alkaline phosphatase, and uric acid levels. Plain radiographs of both knees revealed multiple homogeneous soft tissue calcific opacities distributed periarticularly, without cortical breach, periosteal reaction, or underlying bony erosion (Figures 1A, 1B).

**Figure 2:**
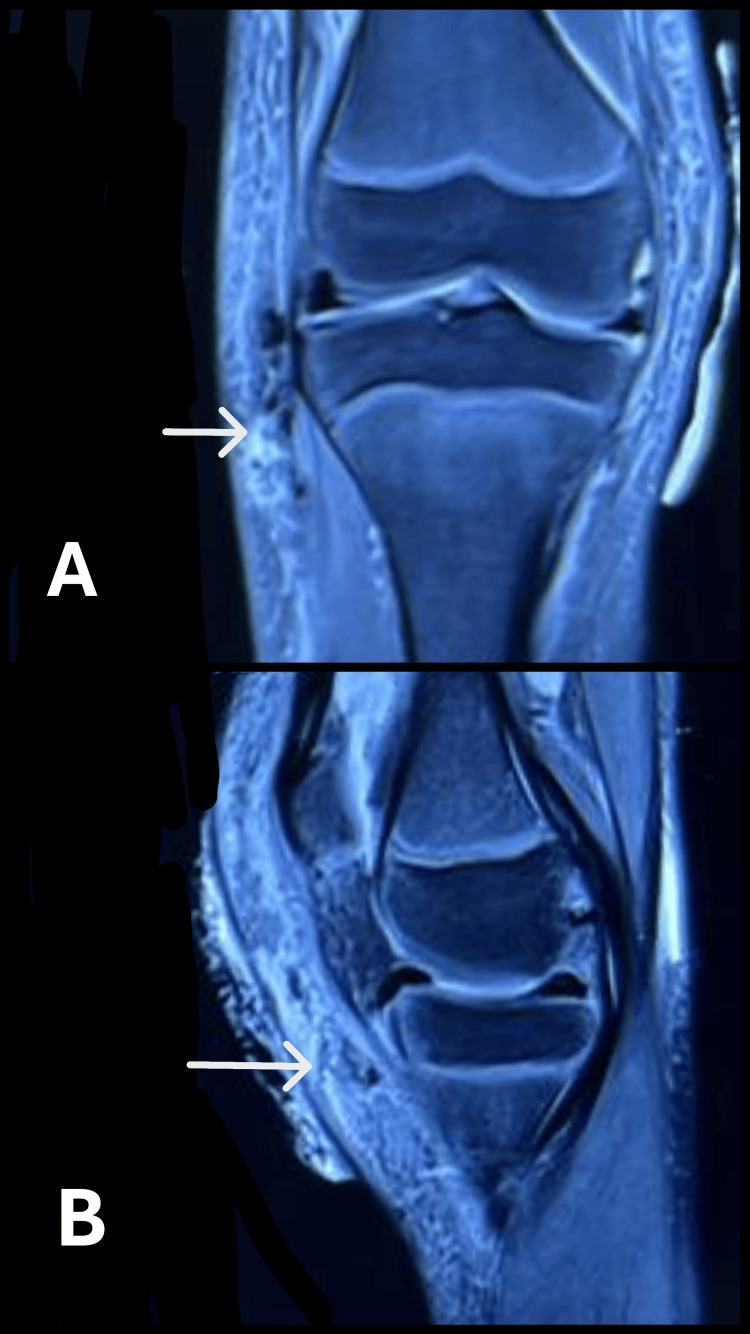
Magnetic resonance imaging of the knee showing calcification (white arrows) (A) Coronal cut. (B) Sagittal cut.

MRI of the right knee demonstrated extensive soft tissue edema in the anterior compartment, with fascial thickening and a fluid collection predominantly in the suprapatellar region (Figure 2). Multiple lobulated calcified masses were noted, appearing hypointense on both T1- and T2-weighted sequences, and surrounded by hyperintense signal changes suggestive of an associated inflammatory response. CT imaging further confirmed the presence of subcutaneous calcifications in the anterior compartment of the knee (Figures 1C, 1D). Ultrasound examination of the neck was unremarkable.

The patient underwent incision and drainage of the thick, viscous fluid collection, along with excision of accessible calcific masses. Adjunctive medical therapy was initiated concurrently. Postoperatively, the knee was immobilized in a posterior slab. Gross examination revealed yellowish-white, pseudoencapsulated masses. Histopathological analysis confirmed the diagnosis, showing irregular calcified deposits surrounded by inflammatory infiltrates composed of neutrophils, lymphocytes, plasma cells, and foreign body-type giant cells within hyalinized fibrocollagenous tissue. Rehabilitation began two weeks after surgery, with gradual knee mobilization and supportive therapy.

## Discussion

Dermatomyositis (DM) is an idiopathic inflammatory myopathy characterized by progressive proximal muscle weakness and distinctive cutaneous manifestations. Tumoral calcinosis is a well-recognized complication, occurring in 20%-40% of JDM cases and more frequently in the juvenile form than in adults. It is typically associated with delayed initiation of immunosuppressive therapy or persistent muscle inflammation. Calcific deposits often develop near pressure-bearing joints and extremities, leading to pain, swelling, and functional impairment [4].

Tumoral calcinosis involves the periarticular deposition of calcium phosphate crystals, forming lobulated, firm masses. Despite its name, it is non-neoplastic and lacks mitotic activity. The pathogenesis is multifactorial, involving metabolic imbalance, autoimmune dysregulation, and chronic inflammation. Based on phosphate levels, it is classified into three subtypes: primary normophosphatemic, primary hyperphosphatemic, and secondary hyperphosphatemic.

In JDM, chronic inflammation alters macrophage function. Normally, macrophages demineralize ectopic calcium via carbonic anhydrase and H⁺-ATPase activity, but in JDM, they become dysfunctional, reducing calcium clearance. Elevated autoantibodies such as anti-NXP2 and PM/Scl can form immune complexes that further damage tissue, a mechanism similar to that in systemic lupus erythematosus [5]. Advanced crystallographic analyses, including synchrotron and X-ray microdiffraction, have shown that these calcific deposits are primarily composed of carbonate apatite, rather than hydroxyapatite or calcium carbonate [6].

Additionally, elevated levels of pro-mineralizing matrix proteins such as bone sialoprotein and dentin matrix protein, along with reduced expression of inhibitory proteins like matrix Gla protein, osteopontin, and fetuin-A, promote ectopic mineralization [7]. Genetic predisposition may also contribute; the TNF-α 308A allele, which is frequently found in JDM patients, is associated with overexpression of TNF-α, a pro-inflammatory cytokine linked to an increased risk of calcinosis.

Experimental models have demonstrated that, in the absence of inflammatory cytokines, osteoclast-like cells can resorb calcific deposits within 28 days. Early mitochondrial calcification following skeletal muscle injury may serve as a nucleation site for crystal deposition [8]. Another emerging mechanism involves neutrophil extracellular trap (NET) formation, where calcium phosphate crystals activate neutrophils, triggering chromatin expulsion and localized inflammation that perpetuates calcification [9]. Clinically, tumoral calcinosis can result in recurrent infections, ulceration, and joint stiffness.

Diagnosis relies on a combination of imaging and laboratory evaluation. CT is particularly useful for defining the extent and morphology of calcific masses, while ultrasound-guided fine-needle aspiration cytology (FNAC) can confirm the diagnosis and, in some cases, provide temporary symptom relief [10]. Ultrasound imaging of the neck may be used to exclude thyroid or parathyroid abnormalities as secondary causes [11]. MRI offers superior soft tissue contrast, aiding differentiation from neoplastic or cystic lesions [12]. In suspected familial forms, genetic testing for mutations in FGF23, GALNT3, or KL is recommended [3].

Management is multifaceted. In hyperphosphatemic subtypes, phosphate-lowering strategies are central and include dietary phosphate restriction and the use of phosphate-binding antacids such as aluminum/magnesium hydroxide [13]. Pharmacologic options include calcium channel blockers (e.g., diltiazem) to reduce intracellular calcium efflux, sodium thiosulfate (topically or intralesionally) as a chelating agent, bisphosphonates to stimulate osteoclastic activity, and probenecid to enhance phosphate excretion [14-17].

Immunosuppressive agents, such as TNF-α inhibitors, rituximab, JAK inhibitors, minocycline, and treprostinil, have shown variable benefit in case series and observational studies [18]. Surgical excision is generally reserved for symptomatic, accessible lesions and tends to be more effective in normophosphatemic patients, although recurrence, infection, and delayed wound healing remain potential complications [1]. Adjunctive measures such as extracorporeal shock wave therapy may also help reduce pain and relieve nerve compression [19].

Future directions

N-acetylcysteine, an antioxidant that has been shown to reduce mitochondrial dysfunction and attenuate type I interferon responses in experimental myositis models, represents a promising potential therapeutic option [20].

## Conclusions

Tumoral calcinosis is a significant and challenging complication of JDM, often linked to prolonged inflammation and delayed initiation of treatment. Its multifactorial pathogenesis, spanning metabolic, genetic, and immune-mediated mechanisms, highlights the importance of early diagnosis using advanced imaging and, when indicated, genetic testing. Effective management requires a multidisciplinary approach that may combine dietary modifications, pharmacologic therapy, and surgical intervention. Emerging treatments, such as antioxidant agents like N-acetylcysteine, offer promise for improving outcomes. Prompt recognition and individualized treatment strategies are key to preventing functional impairment and enhancing quality of life in affected patients.
